# Image quality of spectral brain computed tomography angiography using halved dose of iodine contrast medium

**DOI:** 10.1007/s00234-023-03190-1

**Published:** 2023-07-15

**Authors:** Veronica Fransson, Helena Mellander, Birgitta Ramgren, Henrik Andersson, Francesco Arena, Kristina Ydström, Teresa Ullberg, Johan Wassélius

**Affiliations:** 1 Radiation Physics, Department of Hematology, Oncology and Radiation Physics, Skåne University Hospital, Lund, Sweden; 2 Medical Radiation Physics, Department of Translational Medicine, Lund University, Malmö, Sweden; 3 Department of Medical Imaging and Physiology, Skåne University Hospital, 22185 Lund, Sweden; 4 Department of Clinical Sciences, Lund University, Lund, Sweden; 5 Medical Radiation Physics, Department of Clinical Sciences, Lund University, Lund, Sweden; 6 Neurology, Department of Clinical Sciences Lund, Lund University, Skåne University Hospital, Lund, Sweden

**Keywords:** Computed tomography angiography, Contrast material reduction, Dual-energy CT, Spectral CT, Virtual monoenergetic images, Contrast-induced nephropathy

## Abstract

**Abstract:**

**Purpose:**

Reduction in iodinated contrast medium (CM) dose is highly motivated. Our aim was to evaluate if a 50% reduction of CM, while preserving image quality, is possible in brain CT angiography (CTA) using virtual monoenergetic images (VMI) on spectral CT. As a secondary aim, we evaluated if VMI can salvage examinations with suboptimal CM timing.

**Methods:**

Consecutive patients older than 18 years without intracranial stenosis/occlusion were included. Three imaging protocols were used: group 1, full CM dose; group 2, 50% CM dose suboptimal timing; and group 3, 50% CM dose optimized timing.

Attenuation, noise, signal-to-noise ratio (SNR), and contrast-to-noise ratio (CNR) were measured in the internal carotid artery, M2 segment of the middle cerebral artery, and white matter for conventional images (CI) and VMI (40–200 keV). Qualitative image quality for CI and VMI (50 and 60 keV) was rated by 4 experienced reviewers.

**Results:**

Qualitatively and quantitatively, VMI (40–60 keV) improved image quality within each group. Significantly higher attenuation and CNR was found for group 3 VMI 40–50 keV, with unchanged SNR, compared to group 1 CI. Group 3 VMI 50 keV also received significantly higher rating scores than group 1 CI. Group 2 VMI (40–50 keV) had significantly higher CNR compared to group 3 CI, but the subjective image quality was similar.

**Conclusion:**

VMI of 50 keV with 50% CM dose increases qualitative and quantitative image quality over CI with full CM dose. Using VMI reduces non-diagnostic examinations and may salvage CTA examinations deemed non-diagnostic due to suboptimal timing.

## Introduction

Brain computed tomography angiography (CTA) is an essential tool for vascular assessment. In the imaging workflow of acute stroke patients, CTA is typically combined with non-contrast computed tomography (CT) and CT perfusion and used for the selection and planning of mechanical thrombectomies [1, 2]. This combined imaging examination can be performed within minutes, but CTA and CT perfusion typically require separate injections of iodinated contrast material (CM). For patients undergoing mechanical thrombectomy, additional preoperative CM adds to the total dose administered within less than an hour.

Iodinated CM can cause contrast-induced nephropathy (CIN), a serious complication and the third leading cause of acute kidney injury [3, 4]. CIN is also associated with increased risk of myocardial infarction and neurological complications [5]. The most important risk factors for CIN are pre-existing renal disease, older age, congestive heart failure, diabetes [6–8], and the type and dose of CM [5, 8]. The risk of CIN warrants reduction in CM dose, when possible, especially for risk groups. Iodine CM use also have a negative environmental effect, which further motivates using less CM in clinical practice whenever possible.

Timing of the scan, according to the CM bolus timing, is important for arterial visualization, and if the timing of the arterial phase is suboptimal, it may require the examination to be repeated, increasing both CM dose and radiation dose as well as time. If subjecting the patient to increased CM and radiation dose can be avoided by improving the quality of the original image, it would have great clinical benefit.

During recent decades, CT techniques have been developed to allow reduced CM doses via preserved attenuation from iodine using low kilovolt (kV) X-ray scanning [9, 10] or more recently dual-energy scanning and spectral imaging [11–16]. No study has specifically examined brain CTA, but there are successful examples of CM reduction in other arterial segments without forsaking image quality [11–14]. Spectral CT, dual and multienergy, utilizes the fact that materials have different attenuations at different energies to gain more information about the specific tissue under investigation or to enhance or remove certain materials or structures from the image [17]. Spectral imaging data can be used to reconstruct virtual monoenergetic images (VMI), which represent how the images would have looked if acquired using a chosen monochromatic X-ray source. Low keV VMI amplifies the effect of CM in the image since iodine-based CM has its maximum attenuation at 33 keV [17–19].

The aim of this study was to investigate whether image quality can be preserved in brain CTA with halved CM dose by using VMI reconstructions from a dual-layer detector spectral CT and to investigate whether VMI can compensate for poor arterial enhancement in examinations with suboptimal timing.

## Methods and material

### Study design

This retrospective study was performed to evaluate a clinically initiated optimization project. The study was approved by the Swedish Ethical Review Authority (reference number 2019-02225), and individual informed consent was waived.

### Study population

All patients older than 18 years examined by brain CTA in a dual-layer spectral CT (IQon Spectral CT, Philips Healthcare, The Netherlands) from January to October 2020 were eligible for inclusion in the study. This was a single-site single-system study. Exclusion criteria were as follows: missing spectral data, significant metal artefacts, proximal occlusions affecting the studied arteries, and significant stenosis (>70% in the internal carotid artery). CM administration was followed by a 30-ml saline chases, and bolus tracking with a ROI in the descending aorta set to 130 HU was used to time the scan. The patients were examined by either one of three different imaging protocols used in clinical routine. A patient was assigned to one of the three protocols depending on their examination date, as each protocol was only used for a specific period. Group 1 received full CM dose, group 2 received 50% CM dose with suboptimal timing, and group 3 received 50% CM dose with optimized timing.

Group 1 was examined using the standard imaging protocol with full CM dose (60 ml, Iomeron 400 mg I/ml, Bracco Imaging, Italy). Patients in groups 2 and 3 were examined using a halved CM dose (30 ml). In group 2, the CM dose was halved without compensating the shorter CM bolus by also shortening the scan time, leading to suboptimal contrast timing as time to peak arterial enhancement reduces with smaller volumes of contrast material [20].

For patients in group 3, scan time was corrected and reduced to half that of group 1 to compensate the shortening of the CM bolus and to optimize the scan to the arterial phase.

Patient characteristics (age, sex) as well as radiation dose information (volumetric CT dose index (CTDIvol), dose length product (DLP)) were gathered. Effective doses for patients were calculated as DLP multiplied a conversion factor of 0.0024 mSv/mGycm, according to ICRP 103 weighting factors [21].

### Quantitative image analysis

Quantitative image analysis was performed in the CT manufacturers image viewing software (IntelliSpace Portal 10.1.4.21403, Philips Healthcare, The Netherlands). VMIs were reconstructed using the spectral data files. Quantitative analysis of the image quality was performed for both the conventional image (CI) and VMI images ranging from 40 to 200 keV, in steps of 10 keV. Regions of interest (ROI) were placed in axial images of 2 mm slice thickness images at:

• The terminus of the internal carotid artery (ICA) (1 ± 0.5 mm^2^)

• The dominant M2 segment of the middle cerebral artery (1 ± 0.5 mm^2^)

• The confluence of sinuses (3 ± 1 mm^2^)

• Within homogenous white matter (WM) superior to the lateral ventricles (70–80 mm^2^)

ROI placement was made with special care to avoid partial volume artifacts. Attenuation values (HU) and 1 standard deviation (1SD) were measured for all ROIs. The ICA and M2 ROIs were measured bilaterally, and the average values were used.

Signal-to-noise ratio (SNR) was calculated as $HU/\sqrt{SD}$ for all ROIs. Contrast-to-noise ratio (CNR) was calculated as $\left({HU}_{ROI}-{HU}_{WM}\right)/\sqrt{\left({SD}_{ROI}^2+{SD}_{WM}^2\right)}$ for all arteries relative to white matter. To assess the timing of contrast material in each group, a vein-to-artery ratio was calculated in the CI as the ratio between attenuation values in the confluence of sinuses (CoS) and ICA (*HU*_*cos*_/*HU*_*ICA*_).

### Qualitative image analysis

Qualitative image quality was assessed for both CIs and VMIs of 50 and 60 keV using a visual grading scale. VMIs of 50 and 60 keV were included based on the results of the quantitative image quality analysis. The same reconstructions were used for the qualitative and quantitative analysis (2 mm axial reconstructions). All images were rated by four independent reviewers with a minimum of 5 years of experience reading CTA images (one senior consultant interventional neuroradiologist, one consultant stroke neurologist and two radiologists).

Prior to grading, a joint session was held for all reviewers with a consensus reading of 10 example cases (not cases included in the study) to harmonize grading scales. Visual grading was performed using ViewDEX (Viewer for Digital Evaluation of X-ray images, v. 3.0) [22–24]. ViewDEX presents the images in randomized order, without any image or patient information. Reviewers can freely adjust window settings and zoom. All image grading was performed on an image workstation with dedicated monitors for diagnostic radiology (Coronis^®^ Fusion MDCC-6430 6MP, Barco, Kortrijk, Belgium).

Images were qualitatively graded on four aspects:

1. Overall image quality
2. Visual representation of the internal carotid artery
3. Visual representation of the M2 segment of the middle cerebral artery
4. Timing of the examination

For the first three aspects, a 5-point Likert visual grading scale was used (1 = Non-diagnostic, 2 = Poor, 3 = Fair, 4 = Good, and 5 = Excellent). For the fourth, reviewers could choose between early arterial, late arterial, and venous phases.

### Statistical analysis

#### Analysis of quantitative image quality variables

Mann-Whitney *U* tests were used to compare non-parametric continuous variables and chi^2^ tests were used for comparison of ordinal data. Mann-Whitney *U* tests compared attenuation, CNR, and SNR between the CIs and VMIs. Wilcoxon signed-rank tests were used for intra-group comparisons of CIs and VMIs. *P*-values of less than 0.05 were considered significant. All analyses were performed in RStudio version 1.3.1093 (RStudio Team, Boston, USA).

#### Analysis of virtual grading characteristic data

Rating scores from the visual grading were evaluated using virtual grading characteristic (VGC) analysis in the VGC Analyzer v. 1.0.3 (University of Gothenburg, Gothenburg, Sweden) [25–28]. VGC analysis is a non-parametric rank-invariant method that allows for multiple-reader multiple-case studies. It assesses whether a test condition is superior or inferior to a reference condition. The software produces a VGC curve and calculates the area under the VGC curve (AUC_VGC_) and its uncertainty, represented by a 95% confidence interval, using a resampling technique. An AUC_VGC_ of 0.5 corresponds to equal image quality in the two compared conditions, while an AUC_VGC_ higher than 0.5 means that the test condition was superior, and lower than 0.5 means that the test condition was inferior. For a statistically significant difference between conditions, the value 0.5 cannot be included in the 95% confidence interval. Rating scores were compared both within groups, between CI (reference condition) and VMI (test condition), as well as between groups: Group 1 CI (reference) was compared to group 3 VMI (test), and group 3 CI (reference) was compared to group 2 VMI (test).

The average difference between rating scores for group 1 CI and group 3 VMI, as well as group 3 CI and group 2 VMI, was calculated to appreciate the magnitude of the difference. To determine inter- and intra-rater reliabilities, percentage absolute agreement as well as percentage agreement within one rating scale step were calculated.

## Results

### Patient characteristics

**Fig. 1:**
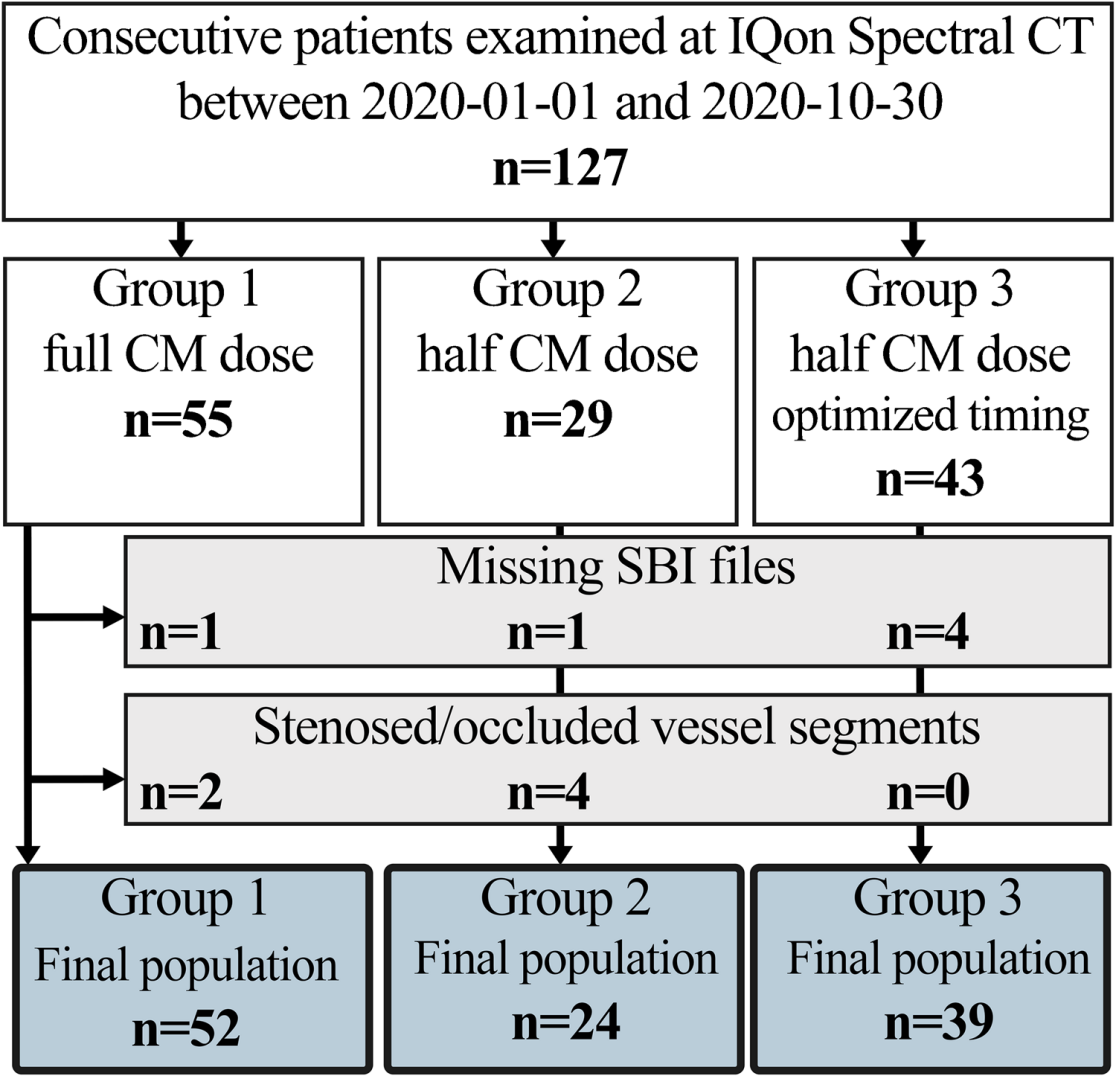
Flow chart presenting the study population, number of excluded/included patients, and reason for exclusion

A total of 127 patients were examined. Of these, 12 patients were excluded based on either intracranial stenosis/occlusions hindering the image analysis (6 patients) or because of missing spectral data files (SBI) (6 patients), resulting in a final study population of 115 patients. Based on the CTA protocol used at the time, 52 patients were in group 1, 29 in group 2, and 43 in group 3. Figure 1 illustrates the study population.

Patient characteristics are presented in Table 1. There were no significant differences in age, sex, CTDI_vol_, DLP, or effective dose between the groups.

**Table 1:** Patient and scanner characteristics for the three groups within the final study population

Variable	Group 1 Full CM dose	Group 2 Half CM dose, suboptimal timing	Group 3 Half CM dose, optimized timing
Clinical parameters			
Number of patients	52	24	39
Men/women	30/22	13/11	16/23
Median age [years] (IQR)	68 (57–75)	75 (52–78)	72 (57–79)
CM dose (400 mg/ml)	60	30	30
Scanner parameters			
Tube voltage [kV]	120	120	120
Collimation	64 × 0.625	64 × 0.625	64 × 0.625
Rotation time [s]	0.50	0.50	0.33
Pitch	0.609	0.609	0.797
Scan time [s]	0.82	0.82	0.41
Median CTDI_vol_ (32 cm) [mGy] (IQR)	12.3 (10.3–14.9)	12.2 (9.6–13.8)	14.6 (10.5–17.0)
Median DLP [mGycm] (IQR)	509 (406–646)	511 (368–566)	586 (466–715)
Median effective dose [mSv] (IQR)	2.5 (2.0–3.2)	2.5 (1.8–2.8)	2.7 (2.3–3.5)

*CM* contrast medium, *IQR* interquartile range, *kV* kilovolt, *CTDI*_*vol*_ volumetric computed tomography dose index (32 cm), *DLP* dose length product, *mGy* milliGray, *mSv* milliSievert

**Fig. 2:**
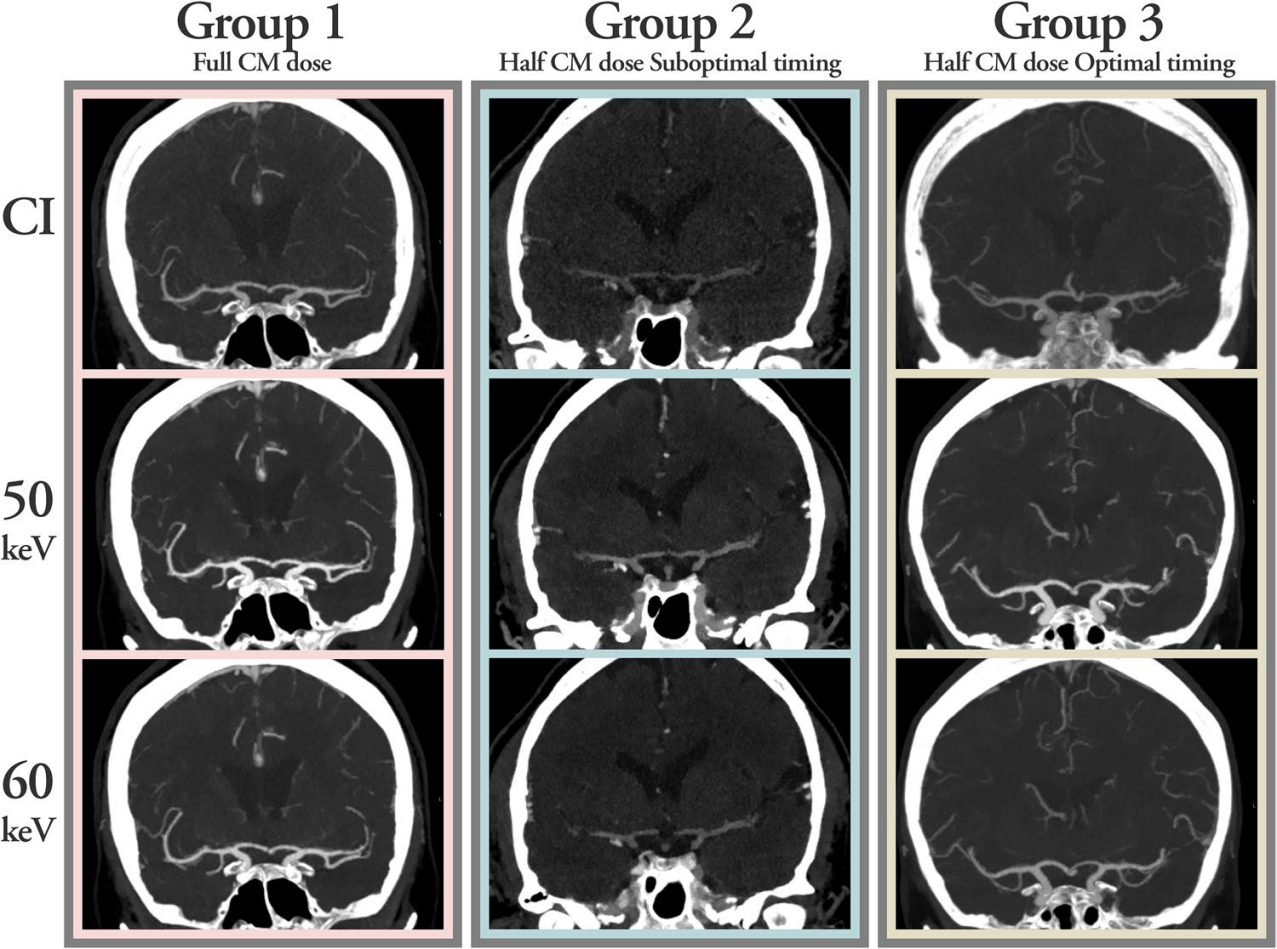
Conventional images (CI) and virtual monoenergetic images (VMI) of 50 and 60 keV for patients in groups 1, 2, and 3

Image examples of CI and VMIs from patients in groups 1, 2, and 3 are shown in Fig. 2.

### Quantitative image quality analysis

**Fig. 3:**
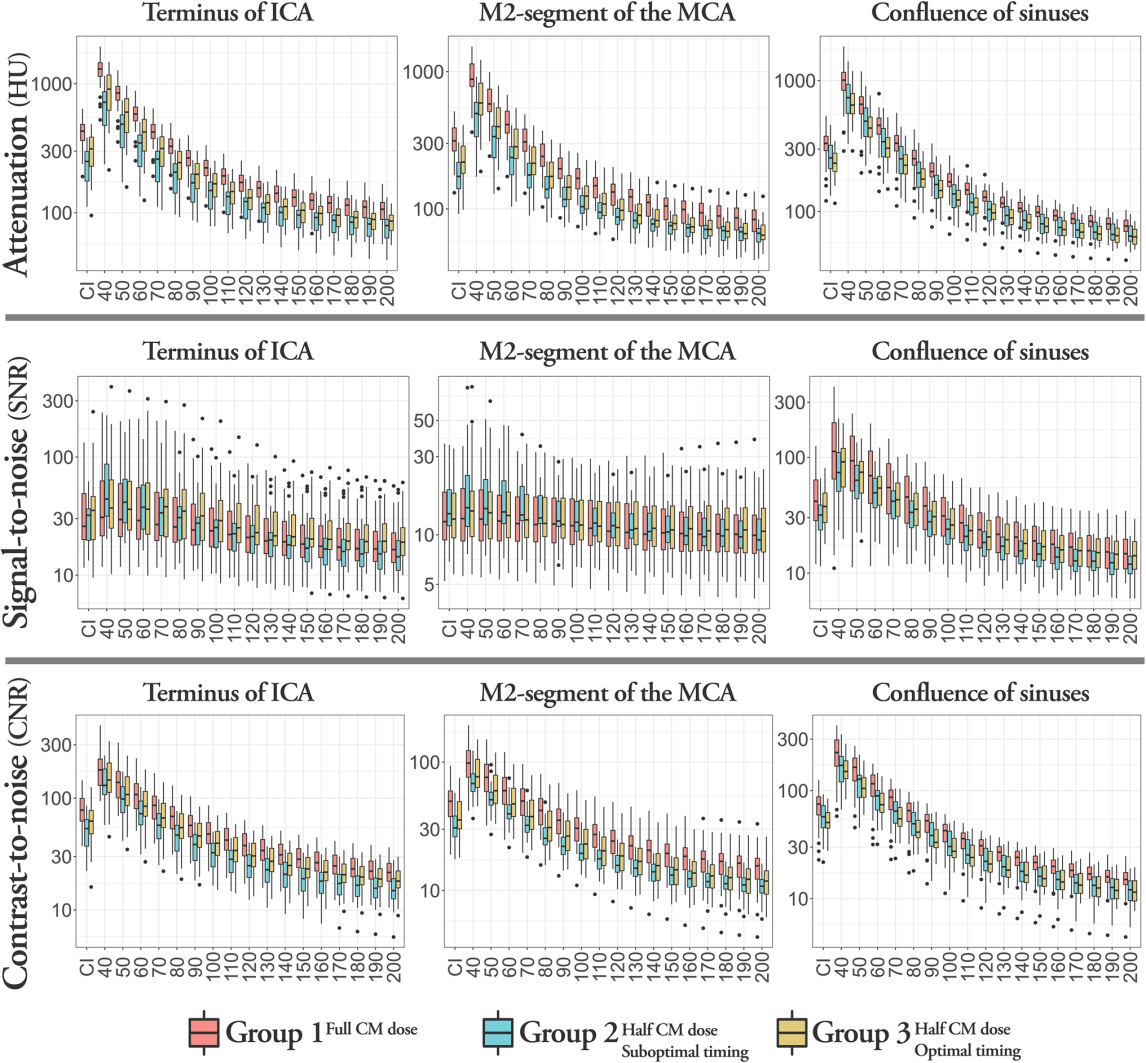
Attenuation values (top row), signal-to-noise ratio (middle row), and contrast-to-noise ratio (bottom row) for the internal carotid artery (ICA) terminus (left column), the M2 segment of the middle cerebral artery (MCA) (middle column), and the confluence of sinuses (right column). Red, group 1; blue, group 2; yellow, group 3

Attenuation values, SNR, and CNR in the predefined ROIs for CI and VMI reconstructions of 40–200 keV are shown in Fig. 3.

### Attenuation values

Distribution of attenuation values in the groups show that group 1, who received full contrast dose, had the highest attenuation of all ROIs and all reconstructions (Fig. 3). Comparing arterial attenuation to attenuation in confluence of sinuses (CoS) in groups 2 and 3, both of which received a halved CM dose, but group 3 also included optimized timing, showed that group 3 had a higher arterial enhancement while group 2 had a higher venous enhancement.

### Signal-to-noise ratio

#### Full contrast dose (group 1 CI) compared to half contrast dose (group 3 VMI)

For ICA, SNR was higher for group 3 VMI of 40–90 keV compared to group 1 CI, but the difference was not statistically significant. However, for group 3 VMIs above 110 keV, SNR was significantly lower. For M2, no statistically significant differences were seen.

#### Half contrast dose-optimized timing (group 3 CI) compared to suboptimal timing (group 2 VMI)

For ICA, SNR was significantly higher for group 2 VMI of 40–60 keV, and significantly lower for VMI higher than 120 keV, compared to group 3 CI. For M2, SNR was higher for VMI of 40–140 keV, but the difference was not statistically significant.

### Contrast-to-noise ratio

#### Full contrast dose (group 1 CI) compared to half contrast dose (group 3 VMI)

For ICA, CNR was significantly higher for group 3 VMI of 40–60 keV compared to group 1 CI. For M2, CNR was significantly higher for VMI of 40 and 50 keV. For both ICA and M2, CNR was significantly lower for VMI of 80–200 keV.

#### Half contrast dose-optimized timing (group 3 CI) compared to suboptimal timing (group 2 VMI)

CNR was higher for group 2 VMI of 40–60 keV in both the ICA and M2 arteries and significantly so for VMI of 40 and 50 keV, compared to group 3 CI.

### Qualitative image analysis

**Fig. 4:**
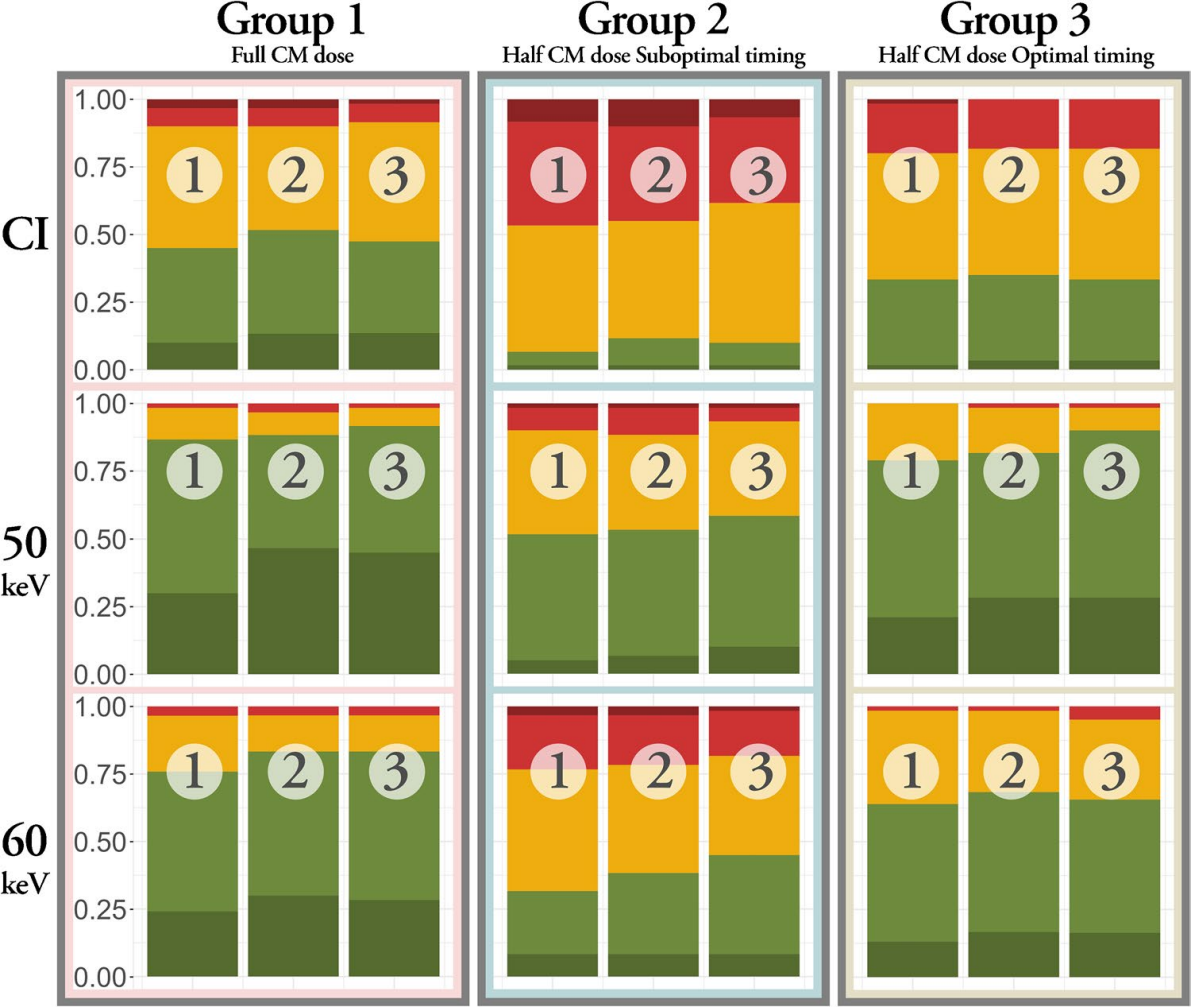
Stacked bar chart for distribution of rating scores for conventional images (CI) and virtual monoenergetic images (VMI). A high rating score of 5 (excellent) is represented in dark green and low rating score of 1 (non-diagnostic) in dark red. Each column is representing one of the three questions: Overall image quality (1); visual representation of the internal carotid artery (ICA) (2) and the M2 segment of the middle cerebral artery (3)

Distributions of the qualitative image quality assessment items are presented in Fig. 4. Within each group, distribution is shifted towards higher rating scores for VMI, compared to CI, with VMI of 50 keV having the highest ratings.

### Virtual grading characteristics analysis

**Fig. 5:**
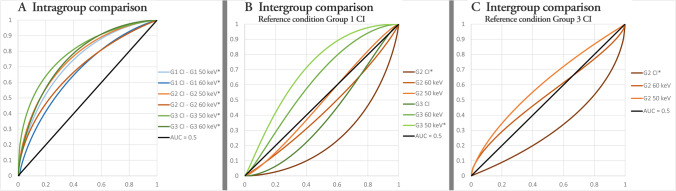
VGC curves for aspect 1 — overall image quality. VGC curve with an AUC_VGC_ of 0.5 is shown in black. Asterisk represents significant AUC_VGC_ . All test conditions received significantly higher scores than their reference condition. **A** Intragroup comparisons with conventional images (CI) as reference condition and virtual monoenergetic images (VMI) as test condition. **B** Intergroup comparisons with reference condition group 1 CI and test conditions group 2 (G2) and group 3 (G3) CI and VMI. **C** Intergroup comparisons with reference condition group 3 CI and test conditions group 2 CI and VMI

VGC curves for the qualitative image quality assessment regarding overall image quality (aspect 1) for intragroup and intergroup comparisons are presented in Fig. 5. AUC_VGC_ (0.5 ≠ 95% CI) higher than 0.5 indicates a higher score for the reference condition, while lower than 0.5 indicates higher scores for the test condition. Within each group, VMIs of both 50 and 60 keV consistently received significantly better ratings than the CIs for all aspects (Fig. 5A). Group 3 VMI of 50 keV received significantly higher ratings on all aspects, compared to group 1 CIs (Fig. 5B). Group 2 CI received significantly lower ratings than both group 1 CI and group 3 CI (Fig. 5B and C). Group 2 VMI did not receive statistically lower ratings than group 1 CI (Fig. 5B).

### Average difference in ratings

#### Full contrast dose (group 1 CI) compared to half contrast dose (group 3 VMI)

On average, group 3 VMI received higher rating scores than group 1 CI, with 0.65 and 0.36 for 50 and 60 keV, respectively.

#### Half contrast dose-optimized timing (group 3 CI) compared to suboptimal timing (group 2 VMI)

On average, group 2 VMI received marginally higher rating scores than group 3 CI, 0.35 and 0.04 for 50 and 60 keV, respectively.

### Contrast timing

The distribution in contrast timing (early arterial, late arterial, or venous phase) according to the qualitative analysis is presented in Table 2 alongside vein-to-artery ratio. Vein-to-artery ratio for group 2 was significantly different from group 1 (*p* < 0.05). There was no significant difference in vein-to-artery ratio between groups 1 and 3 (*p* = 0.52).

**Table 2:** Distribution (percentages) of examinations assessed (average of four independent readers) as early arterial, late arterial, or venous phase as well as vein-to-artery ratio in the three patient groups

Contrast phase assessment	Group 1 Full CM dose	Group 2 Half CM dose, suboptimal timing	Group 3 Half CM dose, optimized timing
Early arterial (%)	7	0	7
Late arterial (%)	87	80	93
Venous (%)	7	20	0
Vein-to-artery ratio	0.77	1.13	0.83

*CM* contrast medium

### Inter- and intra-rater agreements

Percentage agreement, both absolute and within a difference of one rating scale step, is presented in Table 3. For aspects 1–3, inter- and intra-rater percentage absolute agreement was around 50%, but close to 100% when a difference of one rating scale step was permissible.

**Table 3:** Average for all four readers (percentage) for inter- and intra-rater agreements

Image quality assessment question	Inter-rater agreement		Intra-rater agreement	
	Absolute agreement [%]	Agreement within 1 step [%]	Absolute agreement [%]	Agreement within 1 step [%]
1–3	42	90	63	98
4	76	100	88	100

## Discussion

The first aim of this study was to investigate whether the image quality can be preserved in brain CTA with halved CM dose by using VMI reconstructions of spectral data from a dual-layer detector CT.

In our qualitative image quality analysis, use of low keV VMI improved image quality over CI within all three groups. Furthermore, the VMIs had fewer “non-diagnostic” ratings, which may reduce the need for rescans. When comparing VMI with halved contrast dose (group 3) to CI with full contrast dose (group 1), the 50 keV VMIs had significantly higher ratings, suggesting that the image quality is not only preserved but actually improved over CI with full dose of CM. The same tendency was seen also for 60 keV VMIs, but not statistically significant. However, the average difference in rating scores between CI with full CM dose (group 1) and VMI with halved CM dose (group 3) was less than 1 for VMI of 50 keV. Since the Likert scale of 1–5 is chosen to represent meaningful steps in image quality, a difference smaller than 1 step might not be clinically relevant, but we can be confident that VMIs with halved CM dose at least did not perform worse. Another clear benefit is the fewer cases rated “non-diagnostic.”

The results of the qualitative analysis are supported by the quantitative analysis, showing higher attenuation for VMIs at 40–60 keV and higher arterial CNR for VMIs at 40 and 50 keV with halved contrast dose compared to CIs with full contrast dose. Regarding SNR, it was slightly higher for low keV VMIs (below 90 keV) as well as for the halved CM dose compared to CIs with full contrast dose, but the differences were not statistically significant. Our analysis of contrast timing was interpreted as groups 1 and 3 having the same, optimized timing. Thus, detected differences in image quality between the two groups are due to image processing — not timing of examination.

As a secondary aim, we investigated whether VMIs can compensate for poor arterial enhancement in examinations with suboptimal timing.

CIs with suboptimal timing (group 2) were of significantly lower quality than CIs with optimized timing (group 3). Using VMIs, the suboptimally timed group was on par with the optimized group CI. Contrast timing can be suboptimal for different reasons, some of which are unpredictable such as venous stenosis affecting the inflow of injected CM or low cardiac output. Another solution to maximize the chances of acquiring an image of peak arterial attenuation is multiphase CTA, which has the added benefit of collateral assessment [29]. But each additional scan increases radiation dose. By opting to use VMIs, the arterial reproduction may be sufficiently improved without additional imaging.

In this study, we had a group of cases with both suboptimal timing and reduced CM dose. As previously stated, timing issues can occur. And when reducing CM dose in clinical routine, it is important to ascertain that the number of non-diagnostic examinations will not increase. When comparing VMIs of 50 keV with suboptimal timing and 50% CM dose (group 2) to CIs with optimized timing and full CM dose (group 1), we did not find a significant difference or decrease in image quality. These results support that CM dose can be reduced while maintaining image quality, even when taking the risk of obtaining suboptimally timed examinations into consideration.

To the authors’ knowledge, there are no previous studies researching the possibility of contrast reduction in CT angiography of the brain using VMI. There are, however, previous studies on CTA in other arterial segments where they successfully reduced contrast material dose while preserving image quality, using VMI.

In a study on CT aortography using halved CM dose, they compared VMIs at 50 and 77 keV [11]. They found VMIs at 50 keV suitable for vascular assessment, while VMIs at 70 keV, with a noise level comparable to the CI, were suited for nonvascular structure assessment. In our study, we only analyzed the vascular aspects of the image. It is possible that the low keV image should be complemented with a higher keV image for the nonvascular image tasks.

In another study on halved CM dose, investigating CTA of the thoracic aorta showed that VMIs were comparable to CIs with full contrast dose, with 55 keV being the optimal reconstruction (50 keV was not included in the qualitative analysis) [12]. In a study on coronary CTA using halved CM dose, VMIs were able to maintain image quality and there was no significant difference in ability of stenosis (>50%) detection [13]. However, these three studies were all performed using CT systems from different vendors than in our study. A study which was performed on the same kind of CT system as in our study, investigating coronary CTA, showed that CM could be reduced to 40% while maintaining image quality [14]. In our study, we only evaluated the images based on visual appearance and did not include diagnostic tasks for the reviewers. Inclusion of such tasks may increase or decrease the detected difference in image quality between reconstructions.

When there is suspicion of stroke, the patient typically has a CTA even if there is an increased risk of CIN, because the examination is highly motivated. “Neurons over nephrons” CTA should promptly be performed without waiting for creatinine blood testing. But for non-emergency patients, changing the routine and reducing contrast material dosage would have a large impact. It could open the possibility of examining patients that would otherwise not be able to undergo CTA. It is common practice to reduce contrast material dosage for patients with an increased risk of CIN. Reduced contrast material is typically compensated for with a reduction in tube voltage and increased tube loading. Using this method, there is less strain on the patients’ kidneys, and the quality of the examination is maintained without increasing radiation dose. In our study, we investigated a reduction in contrast material in the standard imaging protocol. It would be interesting to test the limits of this method and examine how much further the dosage can be reduced for the high-risk imaging protocol, as these are the patients who stand to gain the most from a reduction. In recent years, photon counting CT has been developed and is in clinical use. Photon counting CT has a potentially higher soft tissue contrast, contrast enhancement, and spatial resolution compared to conventional and dual-energy CT systems. These are all factors which are likely to improve image quality in CTA and enable further reductions in contrast material.

## Conclusion

Using 50% CM dose and 50 keV VMI reconstructions, qualitative and quantitative image quality is greater compared to CIs with full dose of CM. Using VMIs also reduces the number of non-diagnostic examinations and may compensate for suboptimal timing of the arterial phase, thereby salvaging non-diagnostic CTAs.
